# Prevalence of *S. mansoni* Infection and Associated Risk Factors among School Children in Guangua District, Northwest Ethiopia

**DOI:** 10.1155/2022/1005637

**Published:** 2022-04-16

**Authors:** Belaynesh Tazebew, Denekew Temesgen, Mastewal Alehegn, Desalew Salew, Molalign Tarekegn

**Affiliations:** ^1^Department of Biology, College of Science, Bahir Dar University, P.O. Box-79, Bahir Dar, Ethiopia; ^2^Amhara Public Health Institute, Bahir Dar, Ethiopia

## Abstract

**Methods:**

A cross-sectional study design was employed. Four hundred twenty-two participants were selected. Data was collected through observation and interview with structured questionnaire. Stool specimens were collected and examined using two-slide Kato-Katz method. The data were analyzed using SPSS version 23. Logistic regression was fitted for analysis. Variables with *p* value <0.25 in the univariate logistic regression analysis were entered into the multivariable logistic regression model. Those with <0.05 were identified as significantly associated risk factors. To assure the quality of the data, training was given for data collectors and supervisors, and the tools were pretested on 5% of the sample size.

**Results:**

404 (95.7%) school children were enrolled in the study. The overall prevalence of *S. mansoni* was 12.6%. School children in the age group 5-9 years old (AOR (95% CI): 22.27 (3.70-134.01), *p* = 0.001), age group 10-14 years old (AOR (95% CI): 4.58 (1.14-18.42), *p* = 0.032), grade levels 5-8 (AOR (95% CL): 14.95 (4.297-52.03), *p* = 0.001),who swim frequently (AOR (95% CI): 11.35 (2.33-55.33), *p* = 0.003), and those who cultivate near the irrigation area (AOR (95% CI): 7.10 (2.31-21.80), *p* = 0.001) were significantly associated with high risk of *S. mansoni* infection. *Conclusion and Recommendation.* From the finding of the current study, it can be concluded that the prevalence of *Schistosoma mansoni* in the study area is relatively high. Age of fourteen and younger years old, swimming in the river, and irrigation practice were the main risk factors of *S. mansoni* infection. Thus, therapeutic interventions as well as health education are desirable.

## 1. Introduction

*Schistosomiasis* or bilharzia is an acute and chronic parasitic disease caused by a blood fluke (trematode worm) belonging to the genus *Schistosoma* [1]. There are five schistosome species causing the disease, namely, *S. haematobium*, *S. mansoni*, *S. japonicum*, *S. mekongi*, and *S. intercalatum* [2]. The most clinically important species are *Schistosoma mansoni* and *Shistosoma haematobium* [1]*. S. mansoni* causes intestinal schistosomiasis, and it is endemic in sub-Saharan Africa [3]. It is predominant in most parts of the country [4].

In Ethiopia, a number of epidemiological studies showed that intestinal schistosomiasis due to *S. mansoni* infection is widely distributed in several localities of the country with varying magnitudes of prevalence as high as 90% in school children [5]. In Ethiopia, about 5.01 million people are thought to be infected with schistosomiasis and 37.5 million to be at risk of infections [6].

*S. mansoni* infection is transmitted through contact with fresh water polluted with human excreta containing schistosoma eggs, when the egg hatch in fresh water and releasing free swimming miracidia, which infect aquatic snail *Biomphalaria pfeifferi*. *Biomphalaria pfeifferi* is an intermediate host for *S. mansoni* to complete its life cycle and then release cercariae into the water, and human can be infected during contacts with water for various domestic purposes [7, 8]. It is more widespread in poor rural communities particularly in places where fishing and agricultural activities are dominant [9]. Domestic activities such as washing clothes and fetching water from contaminated Rivers/Lakes are the main risk factors for *S. mansoni* infection, which is a potential for children to be infected. Poor hygiene and recreational activities like swimming and fishing also increase the risk of infection in children [10].

In Ethiopia, *S. mansoni* infection is one of the prevalent parasitic diseases reported across many regions, causing considerable morbidity. In northern Ethiopia, in Alamata district, studies revealed 73.9% prevalence of *S. mansoni* infection [11].

Higher rates of infections were observed in males than females due to the frequent water contact behavior of male [12]. Due to they have higher participation in bathing, swimming and irrigation activities [13].

Effective control of the disease requires determining its prevalence rate and identifying risk factors of infection in high-risk population groups [12]. It was important to conduct this research as the study site was appropriate for the study due to the presence of irrigational farming for agricultural product on the river, which is suitable for the intermediate host of *S. mansoni*. Efforts have been made to document the distribution of *S. mansoni* infection nearly at all corners of the region. However, it cannot be said that the distribution of the disease is fully mapped out, as there are recent discoveries of new transmission foci possibly associated with expansion of water development projects and human movement [14].

Studies that indicate the prevalence of *S. mansoni* infection and other intestinal parasites in different areas are crucial for identifying communities at high risk for parasitic infections and for formulating suitable prevention and control measures. The current study aimed to determine the prevalence of *S. mansoni* infection and associated risk factors among school children in two settings of Guangua district, northwest Ethiopia. The findings will help in strengthening the information available so far and encourage policy makers to design effective strategies to combat *S. mansoni* infection.

## 2. Materials and Methods

### 2.1. Study Area and Design

A cross-sectional study was conducted from February to May 2018 to determine the prevalence of *S. mansoni* infection and associated risk factors among school children in Guangua district, northwest Ethiopia. Guangua district is found in Agew Awi Zone, Amhara region. Guangua is bordered on the south and west by the Benshangul-Gumuz region, on the north by Dangla, on the northwest by Faggeta Lekoma and Banja Shekudad, and on the east Ankasha Guagua; the Dura River, a tributary of Abay River, defines parts of its western bordered. The district has 20 rural kebeles and 64 primary schools with an elevation of 1650 m above sea level. The average annual rain fall is 1896.6 mm with an average temperature of 25°C. The total population of the district is 142, 947. The economic base of the majority of the population (93.7%) of the district is agriculture.

### 2.2. Sample Size Determination and Sampling Techniques

A sample size of 422 was determined using single population proportion formula [15]. Proportion of *Schistosoma mansoni* prevalence (*p* = 0.5), level of confidence (*z* = 1.96), and *d* precision (*d* = 0.05) were considered. In addition, 10% nonresponse rate was added.

Multistage sampling was employed to select the study subjects. The study was conducted in Anguay kebele which have two schools. During the study period, the total number of children attending Kibi and Gichgich was 1650 and 921, respectively. The sample size was allocated into two schools based on their total number. This kebele was selected purposively as it is a nearby kebele to the irrigation site in Dura River. The students were stratified by grade level from 1 to 8 in both schools. The numbers of the study participants were selected by systematic sampling technique in each class using their class rosters as sampling frame.

### 2.3. Inclusion and Exclusion Criteria

All school children who gave consent to participate in the study and who had no any history of taking antihelminthes drug during the data collection or with in the last three months were included in the study. While children who were absent on the day of data collection and who did not gave consent were excluded to participate.

### 2.4. Variables

The dependent variable is the *S. mansoni* infection status.

The independent variables were Socio demographic status, water contact habit, defecation practice and latrine availability, shoe wearing habit, irrigation practices, availability of dams, distance between their homes and water bodies, and knowledge about *S. mansoni* infection, its transmission, and prevention method.

### 2.5. Data Collection

#### 2.5.1. Data Collection Tool

A structured interview questionnaire was used to collect data on socio-demographic characteristics and associated risk factors of *S. mansoni* infection. The questionnaire was first developed in English and translated into the local language, Amharic, and then translated back to English to check consistency.

#### 2.5.2. Sample Collection and Examination

A labeled, clean, dry, and leak-proof stool cup was used to collect a stool specimen of about 3 g from each student with an applicator stick, and it was preserved in 10% formalin solution. Then, the stool samples were transported to Bahir Dar University Biomedical Research Laboratory and were processed by Kato-Katz technique, using fixed quantity of sieved 41.7 mg of stool on holed template. They were mounted on slides and covered with malachite green saturated cellophane [9]. Finally, the smeared slides were examined under microscope using 10× and 40× for detection of eggs of the parasite.

### 2.6. Data Analysis

Congregated data were double entered into and analyzed using SPSS version 23 software. Descriptive statistics was carried out to measure relative frequencies and percentages of the variables. Chi-squared tests (*χ*2) were used to determine the association between variables and to test statistical significance differences. Logistic regression analysis was performed to examine associations between variables. Odds ratios (OR) were calculated with 95% confidence interval (CI). Variables having significance at *p* values 0.25 in univariate test were selected for multivariate logistic regression analysis to identify the most important predictors of *Schistosoma mansoni* risk factors based on the test from logistic regression [16]. The associations were considered to be statistically significant when *p* values are less than 0.05.

### 2.7. Data Quality Control

All the necessary reagents, chemicals, and instruments were checked by known positive and negative samples before processing and examination of samples of the study participants. Training was given for data collectors and supervisors. Also the specimens were checked for the serial number, quantity, and procedure of collection. Before the actual data collection, pretest was conducted involving 5% of the sample size that were not part of the sample population in the actual study to ensure the validity of the data collection tool. The smear samples were reexamined by other laboratory experts, which was blinded for the first examination results.

### 2.8. Ethical Considerations

Ethical approval was obtained from the research and community service coordinating office of Science College, Bahir Dar University. Permissions were obtained from school administration/school director office to conduct the study after explaining the purpose and objective of the study. Informed written and oral consent was also obtained from the parent/guardian of the children/their home room teachers. All the data obtained from each study participant was kept confidential. Children found positive for *Schistosoma mansoni* were treated with the WHO standard procedure. Participants who were found infected during the study were provided prescription to take the drug in the nearby pharmacy.

## 3. Results

### 3.1. Socio-Demographic Characteristics of Study Participants

A total of 422 school children were invited to participate, among these, 404 (95.7%) individuals were enrolled in the study, and the remaining 18 individuals were refused to participate. Of the total subject, 227 (56.2%) were male, and 177 (43.8%) were female. The highest proportion of participants 185 (45.8%) were found to be within the age range of 10-14 years. About 269 (66.6%) participants were from grades 1-4, and the remaining 135 (33.4%) were from grade levels 5-8. The parents of majority students (297 (73.5%)) were uneducated (Table 1).

### 3.2. Prevalence of *S. mansoni* Infection

**Figure 1:**
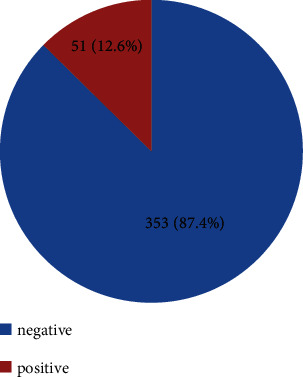
Prevalence of *schistosoma mansoni* infection among school children in Gichgich and Kibi, Guangua district, northwest Ethiopia (2019).

**Figure 2:**
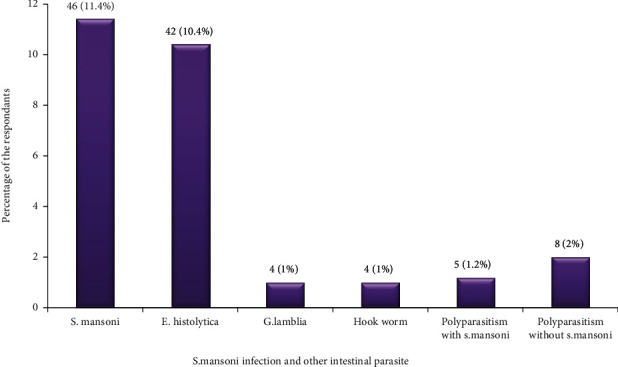
Prevalence of *S. mansoni* infection and other common intestinal parasite among study participants in Anguay kebele, Guangua district, northwest Ethiopia (2018).

The overall prevalence of *S. mansoni* among the study participants was 51 (12.6%), while the remaining 353 (87.4%) were found to be negative for *S. mansoni* infection (Figure 1). In addition to *S. mansoni*, poly parasitism were also observed during the stool samples examination such as *E. histolytica* 42 (10.4%), G. lamblia 4 (1.0%), and Hook worm 4 (1.0%) (Figure 2).

### 3.3. Risk Factors of *S*. *mansoni*

In Chi-square analysis, no statistically significant differences in *S. mansoni* infection were observed among the categories of the variable, age, parent education status, body shower practices in rivers, and knowledge about *S. mansoni* infection and study site. However, the remaining variables were significantly associated with high risk of *S*. *mansoni* infection. *S. mansoni* infection was detected across all categories of the variable with varied prevalence rates of infection. The prevalence of *S*. *mansoni* infection was higher among males 36 (15.9%) than females 15 (8.5%) participants. The prevalence of *S. mansoni* infection was higher 28 (15.1%) among the study participants in the age group 10-14 years followed by age group of 5-9 years 16 (12.2%) and 15-19 years 7(8.0%). Students who attend grades 5-8 (22.2%) were highly infected by *S*. *mansoni* compared to those grades 1-4 (7.8%) (Table 2).

The majority of 304 (75.2%) participants washes their clothes and utensils in the River, amongst those 45 (14.8%) were positive for *S. mansoni* infection. With regard to swimming habits in the rivers, the prevalence of *S*. *mansoni* infection was higher in those who swim always 29 (38.2%) and followed by some times 18 (6.8%) and not at all 4 (6.3%). In this study, respondents who cross the water bodies (37 (22.6%)), who were involved at irrigation practices (43 (22.8%)), and who live at areas where there are dams in their locality (28 (17.4%)) were found to be positive for *S. mansoni* infection. In terms of distance between their homes and water bodies, the highest prevalence was observed in near distance (<1 KM) 37 (18.6%) compared to far distance (≥1 KM) 14 (6.8%). School children who had no the habit of shoe wearing (38 (16.3%)) and absence of latrine in their homes (42 (17.1%)) were more infected with *S. mansoni* (Table 2).

This result showed that more than half (253 (62.6%)) of the study participants did not know about the disease, *S. mansoni*, and its transmission methods among these (41 (16.2%)) were positive. Sixty one percent of study participants had no knowledge about the burden of *S. mansoni* infection, of those majority (42 (16.7%)) were affected. More than half (250 (61.9%)) of the respondents defecate in open field. Among *S. mansoni* positive individuals, majority of them were defecates in open field (43(17.2%)) compared to those who did not defecate in open field (8 (5.2%)) (Table 2).

### 3.4. Multivariate Analyses of *S. mansoni* Infection and Its Associated Factors

In multivariate analysis, the significant independent predictors of *S. mansoni* infection in this study were the age group 5-9 years and age group 10-14 years, grade level 5-8, always swimming in the River, having irrigation practice, and crossing the water bodies, while the remaining variables were not observed to have any significant association with *S. mansoni* infection (Table 3).

In this study, the likelihood of *S. mansoni* infection among participants who belonged to 5-9 years old age group was significant and about 22 times higher (AOR = 22.27, 95% CI 3.70-134.01, *p* = 0.001). The odds of *S. mansoni* infection were also significantly four times higher risk in age group of 10-14 years (AOR = 4.58, 95% CI 1.14-18.42, *p* = 0.032). Regarding the grade level of the study participants, grades 5-8 were about fifteen times at higher risk of *S. mansoni* infection than those who enrolled in grade levels 1-4, and it was statistically significant (AOR = 14.95, 95% 4.297-52.03, *p* = 0.001) (Table 3).

The odds of positive *S. mansoni* infection were significantly eleven times higher among individuals swimming in the river always compared to never swimming in the River at all (AOR = 11.35, 95% CI 2.33-55.33, *p* = 0.003). Subjects who practice irrigation were seven times positively associated with *S. mansoni* infection (AOR = 7.10, 95% CI 2.31-21.80, *p* = 0.001) (Table 3).

## 4. Discussion

The overall prevalence rate of *S. mansoni* among study participants in the current study was 12.6%. It is comparable with the study finding in an endemic area of Niger River basin (12.5%) [17] and Agaie, Niger State (10.17%) [18] of Nigeria, and Dembia (15.4%) [19] and (11.4%) Wondo District of Ethiopia [20].

However, it was higher than the prevalence among preschool children in Gondar town (5.9%) [21], among the school children in Côte d'Ivoire (6.1%) [22], and in the White Nile River Basin of Sudan (5.9%) [23]. Higher prevalence of *S. mansoni* in the current study could be due to the existence of Dura River where the local community uses for washing clothes, taking baths, and fetching water for domestic purpose. The river may serve as a potential source of infection for *S. mansoni*. Moreover, the weather condition in the area is relatively warmer and more humid which favor the existence and reproduction rate of the snail. The other possible cause might be related with the differences in children's behavior to water contact and level of awareness about the prevention and control of *S. mansoni* infection.

The prevalence in the current study was lower than the overall prevalence of *S. mansoni* infection among school children in districts of north Gondar (Chuahit, Sanja, Debark, and Maksegnit) (33.5%) [24], nearby rivers in Jimma town (28.7%) [25], in rural area of Bahir Dar (24.9%) [26], in Jimma Zone (27.6%) [27], southern Ethiopia (25.8%) [28], and Mekelle city (23.9%) [12]. This study was also much lower as compared to the finding in Sanja area, Amhara region (82.8%) [29], and Damot Woide district, Wolaita Zone (81.3%) [14]. The variations in the prevalence may be due to some factors such as difference in water contact habit, toilet utilization, and ecological distribution of snails in the study area (the presence of fast running rivers (Dura) in the current study may lead to low availability of vector snails, since snails mainly prefer stagnant or slow-moving water bodies). The snail (*Biomphalaria pfeifferi*), responsible for the transmission of *S. mansoni* infection, is more prevalent in areas 2000 m above sea level [30]. The variations in the prevalence in this study might also be due to factors which are related with the characteristics of intermediate snail hosts.

In the current study, polyparasitism was observed other than the *S. mansoni* infection. Even if they perform sanitation; it is not enough in preventing schistosoma infection [31]. On the other hand, *S. mansoni* and other parasite infections will never be a public health problem if there are appropriate improvements of hygiene and sanitation standards [32]. The presence of adequate sanitation does not necessarily guarantee its use. The bulk of *Schistosoma* specious eggs reach directly into the water usually by children during bathing and swimming. The use of adequate sanitation systems for urine and faeces has reduced schistosomiasis within short periods of time, while it takes longer for other helminths such as *Ascaris lumbricoides* and *Trichuris trichiura* [31].

Though a study in two the settings of Côte d'Ivoire [22] reported similar prevalence rates for boys and girls in Kenya [33], many studies showed contrasting finding in the prevalence of *S. mansoni* infection by sex [32–34]. The finding of the current study also showed higher prevalence of *S. mansoni* infection in males than in female. It is in agreement with the studies done in Wolaita Zone of Ethiopia [14] and the findings in Niger [35] and central Sudan [36]. Male children may have higher exposure of contact with cercariae contaminated water bodies than females while helping their family in outdoor activities such as herding cattle.

Unlike a study in Sanja area of Amhara, Ethiopia [29], which reported a similar prevalence of *S. mansoni* across the age group of school children, the current finding showed that students in the age group of 14 years old and younger were at a higher risk of acquiring *S. mansoni* infection than those with the age range of 15-19. It was in line with previous studies done in Ethiopia [29, 36]. This might be due to their chance of playing in the field which increases the probability of contact with cercaria contaminated water bodies.

In the current study, the prevalence of *S. mansoni* infection was significantly associated with the frequency of swimming in the river. The chances of positive *S. mansoni* infection were eleven times higher in individuals who always swim in the river compared to those who never swim. This was in agreement with studies done in Hawassa and Gorgora of Ethiopia [33, 37].

The current study revealed that water contact habit through irrigation practice and crossing the water bodies in their way to school are associated with high prevalence of *S. mansoni* infection; this was in line with the study at Wolaita Zone, Southern [14] and Gondar town of Ethiopia [21], and south Côte d'Ivoire, and central Côte d'Ivoire [38], which reported that water contact at stream crosses and herding cattle near the stream increase the risk of *S. mansoni* infection.

This study also showed that working in an irrigated field is significantly associated with *S. mansoni* infection. This is in agreement with the findings of the studies at Dudicha and Shesha Kekel localities [39], with different water source users in Tigray region of Ethiopia [40] and southeastern Brazil [41]. Distance from the water bodies was also associated with *S. mansoni* infection. Children who live near to the water bodies were more infected with *S. mansoni* than those who live far. It was in agreement with previous findings of a study in Cote d'Ivoire [42]. This study also showed that the prevalence of *S. mansoni* infection was associated with shoe wearing habit. This is in line with the findings of a study in Jiga town [43]. This could be due to the agricultural-based economy of the community; closer distance with water bodies and continuous barefoot water contact inhabit increase the chance of contact with water bodies that contain *S. mansoni* cercariae.

## 5. Conclusion and Recommendation

*S. mansoni* infection was the major problem among school children in the current study. Students having fourteen and younger years old, students who frequently swim in the river, having irrigation practice, and cross the river bodies were at a higher risk of *S. mansoni* infection. Hence, it is recommended to focus on raising the awareness of the school children about the prevention and control measures of *S. mansoni* in the locality. In addition, responsible bodies on irrigational practices should work on the regular cleaning of water canals which favor the reproduction of the snail.

## Figures and Tables

**Table 1:** Socio-demographic characteristics of the study participants study participants in Gichgich and Kibi, Guangua district, northwest Ethiopia (2019).

Variable	Category	Frequency	Percent
Sex	Male	227	56.2
	Female	177	43.8
Age	5-9	131	32.4
	10-14	185	45.8
	15-19	88	21.8
Grade level	1-4	269	66.6
	5-8	135	33.4
Parent education status	Uneducated	297	73.5
	Educated	107	26.5

**Table 2:** Chi-square analysis of association of *Schistosoma mansoni* infection with socio demographic and associated risk factors of study participants in Gichgich and Kibi, Guangua district, northwest Ethiopia (2019).

Variables	Category	Number examined *n* (%)	*S. mansoni* infection		*χ*2, *p*
			Positive	Negative	
Sex	Male	227 (56.2)	36 (15.9)	191 (84.1)	4.917, 0.027
	Female	177 (43.8)	15 (8.5)	162 (91.5)	
Age	5-9	131 (32.4)	16 (12.2)	115 (87.8)	2.817, 0.244
	10-14	185 (45.8)	28 (15.1)	157 (84.9)	
	15-19	88 (21.8)	7 (8.0)	81 (92.0)	
Grade level	1-4	269 (66.6)	21 (7.8)	248 (92.2)	16.935,0.001
	5-8	135 (33.4)	30 (22.2)	105 (77.8)	
Parent education status	Uneducated	254 (62.9)	43 (14.5)	254 (85.5)	3.496, 0.062
	Educated	107 (73.5)	8 (7.5)	99 (92.5)	
Washing clothes and utensils in the river	Yes	304 (75.2)	45 (14.8)	259 (85.2)	5.286, 0.021
	No	100 (24.8)	6 (6.0)	94 (94.0)	
Frequency of swimming in the rivers	Always	76 (18.8)	29 (38.2)	47 (61.8)	55.348, 0.001
	Some times	264 (65.3)	18 (6.8)	246 (93.2)	
	Not at all	64 (15.8)	4 (6.3)	60 (93.8)	
Body shower practices in in rivers	Yes	332 (82.2)	42 (12.7)	290 (87.3)	0.001, 0.972
	No	72 (17.8)	9 (12.5)	63 (87.5)	
Crossing the water bodies by the respondents' way to and from school	Yes	164 (40.6)	37 (22.6)	127 (77.4)	24.715, 0.001
	No	240 (59.4)	14 (5.8)	226 (94.2)	
Irrigation practices	Yes	189 (46.8)	43 (22.8)	146 (77.2)	33.024, 0.001
	No	215 (53.2)	8 (3.7)	207 (96.3)	
Presence of dams in their locality	Yes	161 (39.9)	28 (17.4)	133 (82.6)	5.516, 0.019
	No	243 (60.1)	23 (9.5)	220 (90.5)	
The distance between their homes and water bodies	Near (<1 KM)	199 (49.3)	37 (18.6)	162 (81.4)	12.669, 0.001
	Far (≥1 KM)	205 (50.7)	14 (6.8)	191 (93.2)	
Shoe wearing habits	Yes	171 (42.3)	13 (7.6)	158 (92.4)	6.778, 0.009
	No	233 (57.7)	38 (16.3)	195 (83.7)	
Presence of latrine in their homes	Yes	159 (39.4)	9 (5.7)	150 (94.3)	11.526, 0.001
	No	245 (60.6)	42 (17.1)	203 (82.9)	
Open defecation	Yes	250 (61.9)	43 (17.2)	207 (82.8)	12.452, 0.001
	No	154 (38.1)	8 (5.2)	146 (94.8)	
Knowledge about *S. mansoni* infection and its transmission method	Yes	151 (37.4)	10 (6.6)	141 (93.4)	7.873, 0.005
	No	253 (62.6)	41 (16.2)	212 (83.8)	
Knowledge about the burden of *S. mansoni* infection in their area	Yes	153 (37.9)	9 (5.9)	144 (94.1)	10.147, 0.001
	No	251 (62.1)	42 (16.7)	209 (83.3)	
Knowledge about *S. mansoni* infection prevention	Yes	160 (39.6)	14 (8.8)	146 (91.3)	3.604, 0.058
	No	244 (60.4)	37 (15.2)	207 (84.8)	
Study site	Gichgich	201 (49.8)	29 (14.4)	172 (85.6)	1.180, 0.277
	Kibi	203 (50.2)	22 (10.8)	181 (89.2)	

Statistically significant at *p* < 0.05.

**Table 3:** Multivariate logistic regression analysis of *Schistosoma mansoni* prevalence with selected seemingly significant variables in Gichgich and Kibi, Guangua district, northwest Ethiopia (2019).

List of variable	Category	*S. mansoni* infections		COR (95% CI)	*p* value	AOR (95% CI)	*p* value
		Positive	Negative				
Sex	Male	36 (15.9)	191 (84.1)	2.04 (1.08, 3.85)	0.029	1.48 (0.574, 3.84)	0.416
	Female	15 (8.5)	162 (91.5)	1.00		1.00	

Age	5-9	16 (12.2)	115 (87.8)	1.61 (0.63, 4.09)	0.317	22.27 (3.70,134.01)	0.001∗
	10-14	28 (15.1)	157 (84.9)	2.06 (0.86, 4.93)	0.103	4.58 (1.14, 18.42)	0.032∗
	15-19	7 (8.0)	81 (92.0)	1.00		1.00	

Grade level	5-8	21 (7.8)	248 (92.2)	3.37 (1.85, 6.16)	0.001	14.95 (4.297,52.03)	0.001∗
	1-4	30 (22.2)	105 (77.8)	1.00		1.00	

Parent education status	Uneducated	43 (14.5)	254 (85.5)	2.30 (0.95, 4.61)	0.070	2.77 (0.96, 7.96)	0.059
	Educated	8 (7.5)	99 (92.5)	1.00		1.00	

Washing clothes and utensils in the river	Yes	45 (14.8)	259 (85.2)	2.72 (1.13, 6.59)	0.026	2.50 (0.71, 8.87)	0.156
	No	6 (6.0)	94 (94.0)	1.00		1.00	

Frequency of swimming in the rivers	Always	29 (38.2)	47 (61.8)	9.26 (3.04,28.17)	0.001	11.35 (2.33, 55.33)	0.003∗
	Some times	18 (6.8)	246 (93.2)	1.10 (0.36, 3.36)	0.870	1.48 (0.33, 6.70)	0.613
	Not at all	4 (6.3)	60 (93.8)	1.00		1.00	

Crossing the water bodies by the respondents' way to and from school	Yes	37 (22.6)	127 (77.4)	4.70 (2.45, 9.03)	0.001	4.17 (1.70, 10.21)	0.002∗
	No	14 (5.8)	226 (94.2)	1.00		1.00	

Irrigation practices	Yes	43 (22.8)	146 (77.2)	7.62 (3.48,16.68)	0.001	7.10 (2.31, 21.80)	0.001∗
	No	8 (3.7)	207 (96.3)	1.00		1.00	

Presence of dams in their locality	Yes	28 (17.4)	133 (82.6)	2.01 (1.11, 3.64)	0.020	0.67 (0.25, 1.77)	0.417
	No	23 (9.5)	220 (90.5)	1.00		1.00	

The distance between their homes and water bodies	Near (<1 KM)	37 (18.6)	162 (81.4)	3.12 (1.63, 5.97)	0.001	1.67 (0.63, 4.44)	0.303
	Far (≥1 KM)	14 (6.8)	191 (93.2)	1.00		1.00	

Shoe wearing habits	Yes	13 (7.6)	158 (92.4)	0.42 (0.22, 0.82)	0.011	0.41 (0.16, 1.80)	0.076
	No	38 (16.3)	195 (83.7)	1.00		1.00	

Presence of latrine in their homes	Yes	9 (5.7)	150 (94.3)	0.29 (0.14, 0.61)	0.001	0.33 (0.06, 1.84)	0.204
	No	42 (17.1)	203 (82.9)	1.00		1.00	

Open defecation	Yes	43 (17.2)	207 (82.8)	3.79 (1.73, 8.30)	0.001	2.39 (0.41, 13.90)	0.333
	No	8 (5.2)	146 (94.8)	1.00		1.00	

Knowledge about *S. mansoni* infection and its transmission method	Yes	10 (6.6)	141 (93.4)	0.37 (0.18, 0.76)	0.007	0.70 (0.26, 1.90)	0.485
	No	41 (16.2)	212 (83.8)	1.00		1.00	

Knowledge about the burden of *S. mansoni* infection in their area	Yes	9 (5.9)	144 (94.1)	0.31 (0.15, 0.66)	0.002	0.391 (0.14, 1.06)	0.064
	No	42 (16.7)	209 (83.3)	1.00		1.00	

Knowledge about *S. mansoni* infection prevention	Yes	14 (8.8)	146 (91.3)	0.54 (0.28, 1.03)	0.061	0.46 (0.18, 1.18)	0.106
	No	37 (15.2)	207 (84.8)	1.00		1.00	

COR = crude odd ratio; sig. at *p* ≤ 0.25; AOR∗ = adjusted odd ratio; sig. at *p* ≤ 0.05.

## Data Availability

The datasets used and analyzed during the current study are available from the corresponding author on reasonable request (in SPSS code).

## Abbreviations

OR: Odd ratio
AOR: Adjusted odds ratio
COR: Crude odds ratio
CI: Confidence interval
ETB: Ethiopian birr
KM: Kilometer
*S. mansoni*: *Schistosoma mansoni*
*S. haematobium*: *Schistosoma haematobium*
*S. japonicum*: *Schistosoma japonicum*
*S. mekongi*: *Schistosoma mekongi*
*S. intercalatum*: *Schistosoma intercalatum*
*E. histolytica*: *Entameoba histolytica*
G.lamblia: Gardia lamblia
H. worm: Hook worm.
